# Archaeal DNA Repair Mechanisms

**DOI:** 10.3390/biom10111472

**Published:** 2020-10-23

**Authors:** Craig J. Marshall, Thomas J. Santangelo

**Affiliations:** Department of Biochemistry and Molecular Biology, Colorado State University, Fort Collins, CO 80523, USA; c.marshall@colostate.edu

**Keywords:** archaea, DNA repair, DNA metabolism, genomic integrity, double-strand breaks, DNA modifications, DNA damage

## Abstract

Archaea often thrive in environmental extremes, enduring levels of heat, pressure, salinity, pH, and radiation that prove intolerable to most life. Many environmental extremes raise the propensity for DNA damaging events and thus, impact DNA stability, placing greater reliance on molecular mechanisms that recognize DNA damage and initiate accurate repair. Archaea can presumably prosper in harsh and DNA-damaging environments in part due to robust DNA repair pathways but surprisingly, no DNA repair pathways unique to Archaea have been described. Here, we review the most recent advances in our understanding of archaeal DNA repair. We summarize DNA damage types and their consequences, their recognition by host enzymes, and how the collective activities of many DNA repair pathways maintain archaeal genomic integrity.

## 1. Introduction

Aside from the intrinsic instability of DNA, genomes are threatened by a plethora of endogenous and exogenous insults. Left unrepaired, DNA damage increases mutation rates, causing adverse effects on cellular health, with often drastic consequences to cellular and organismal fitness. Endogenous damage has many sources: genomic material can spontaneously undergo base hydrolysis or deaminate, and torsional stresses brought about by information processing systems can bring about genomic instability. Cellular machineries will occasionally incorporate mismatch errors or ribonucleotide monophosphates (rNMPs) into newly synthesized DNA, and many metabolic enzymes produce reactive oxygen species (ROS) which may oxidize DNA bases. Cells must also tolerate exogenous sources of DNA damage which vary depending on the external environment. Chemical crosslinkers, environmentally generated ROS, ultraviolet light, and ionizing radiations, from within or which penetrate the atmosphere, all have mutagenic effects on DNA.

**Figure 1:**
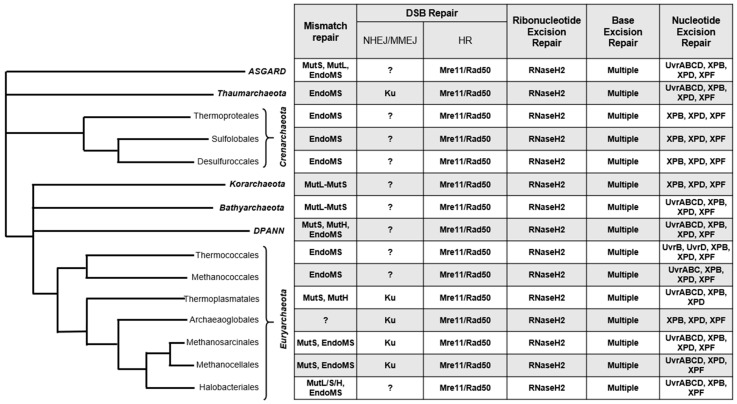
Predicted distribution of pathway-specific archaeal DNA repair proteins by clade [10], according to KEGG (Kyoto Encyclopedia of Genes and Genomes) orthologies. Many pathways appear conserved, with most variation found in distribution of mismatch repair (MMR) and nucleotide excision repair (NER) proteins.

Many archaea thrive within niche and extreme environments which can increase rates of DNA damage. Many halophilic archaea, for example, thrive in shallow salt plains and endure extreme levels of UV radiation [1], while some hyperthermophilic species persist at temperatures that would easily denature purified DNA [2,3], and yet, the presumed increased rates of deamination, depurination, and oxidation are somehow tolerated [4,5,6]. In addition to growth in the extremes, many archaeal species maintain genomic stability levels to display similar rates of spontaneous mutation to mesophilic prokaryotes such as *Escherichia coli* [7,8,9]. Perhaps surprisingly, no unique DNA repair pathways have been described in Archaea (Figure 1), nor extremophilic Bacteria, i.e., *Deinococcus radiodurans*. Insight into how Archaea detect and convert damaged DNA bases into repairable substrates has begun to reveal how genomic integrity is preserved in extremis. Here, we review current knowledge of archaeal DNA repair pathways and examine both discrepancies and outstanding questions in the field.

**Figure 2:**
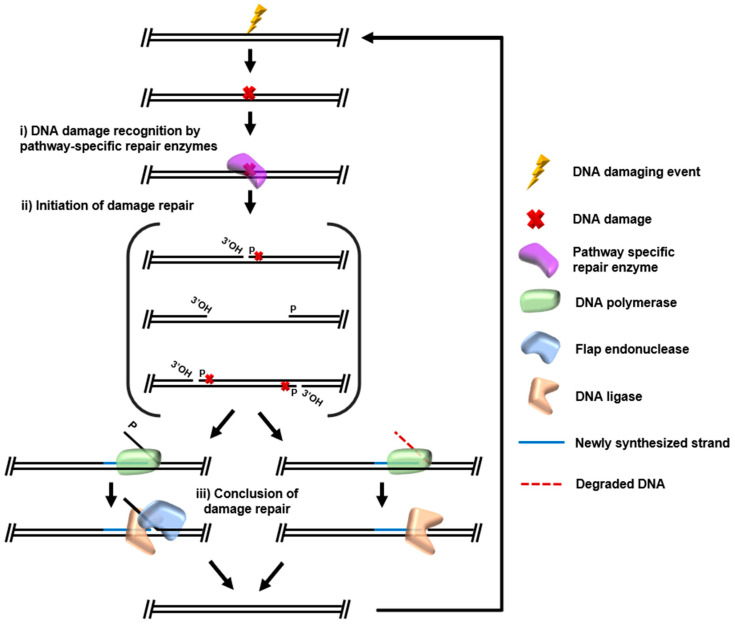
Archaeal DNA repair pathways follow similar generalized steps: (i) Recognition of DNA damage by pathway-specific enzymes. (ii) Initiation of repair by conversion of DNA damage into appropriate and repairable substrate. (iii) Conclusion of repair by resynthesis of damaged DNA from a complementary undamaged strand, degradation of damaged strand by flap endonuclease of intrinsic DNA polymerase exonuclease activity, and nick ligation by DNA ligase.

A multitude of strategies to identify modified nucleotides or damaged DNA structures (here, collectively termed recognition) and initiate repair is encoded in most genomes, with processes for recognition and repair perhaps best studied within mesophilic bacteria and eukarya. Some DNA damage repair can be directly reversed, i.e., photoreactivation of thymine–thymine dimers by photolyases and repair of methylation adducts by alkyltransferases such as AlkB [11,12]. However, DNA repair more commonly involves pathways which require several specialized enzymes through steps of damage recognition, initiation of repair, and final polymerization/ligation of resynthesized DNA. Collectively, the cycle of recognition-, initiation-, and ligation-based DNA repair (Figure 2) dominates the conserved DNA repair pathways that account for the majority of DNA repair, be it double-strand break (DSB) repair, mismatch repair (MMR), ribonucleotide excision repair (RER), base excision repair (BER), or both global genomic and transcription coupled nucleotide excision repair (GG-NER, TC-NER). The core DNA repair pathways generally consist of recognition factors that more often than not cleave the DNA backbone and or glycosidic linkage to the nucleotide base, a repair DNA polymerase (DNAP) for strand resynthesis, a nuclease (or the exonuclease activity of DNAP) for removal of damaged bases/strands displaced during resynthesis, and DNA ligase to seal nicks generated during repair.

## 2. Double-Strand Break (DSB) Repair

DSBs are potentially the most mutagenic of all DNA damaging events. As the name suggests, DSBs involve a co-localized break in the phosphodiester backbones of both DNA strands, permitting regions of the genome to separate and offering the potential that the wrong ends, or trimmed ends of the DNA will be linked with the loss or repositioning of genetic information. DSBs can be generated “accidentally” by missteps of information processing machineries, i.e., by mistiming of replication, replication–transcription complex conflicts, and replication or transcription through existing DNA damage/secondary structures [13,14,15,16]. DSBs are also purposefully generated as essential intermediates of many nucleic acid metabolism pathways [17,18,19,20] and if such pathways are aborted prematurely, intermediate complexes may be released inappropriately. Unchecked DSBs can be extremely detrimental to cellular health, causing arrests of replication and transcription which may lead to apoptosis. The potential cytotoxicity of DSBs necessitates their repair, and thus, multiple conserved pathways (both homologous recombination (HR)-based and error-prone) have evolved in cells to restore functional genomic architecture.

### 2.1. Error-Prone DSB Repair Pathways

It is likely, especially in Archaea with low or varying ploidy [21,22], that HR is not always a readily available pathway for the efficient repair of DSBs. This is highlighted by the evolution of alternative methods of DSB repair which do not require an undamaged template strand for repair. Two conserved DSB repair methods—Microhomology-Mediated End Joining (MMEJ) and Non-Homologous End Joining (NHEJ) [23,24]—are relatively rapid and simple but both pathways are prone to loss of genetic material.

**Figure 3:**
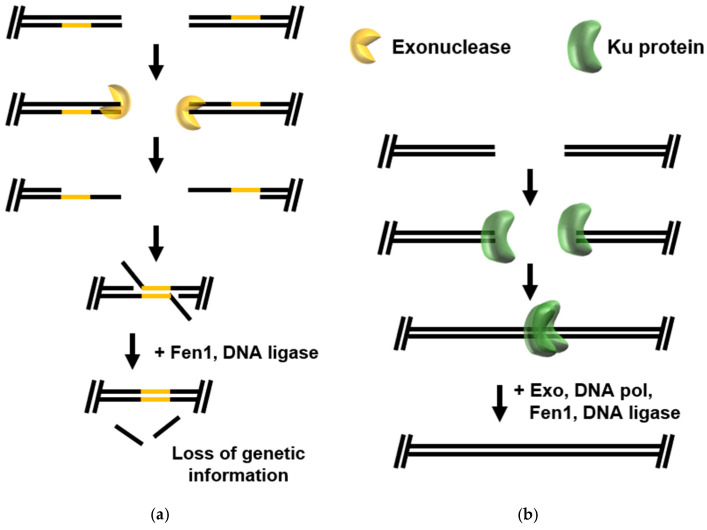
“Error-prone” double-strand break (DSB) repair pathways in Archaea: (**a**) In microhomology-mediated end joining (MMEJ), small regions of microhomology (yellow) are revealed by exonuclease activity; annealing and subsequent processing by flap endonuclease and DNA ligase often results in the loss of genetic information. (**b**) Non-homologous end joining (NHEJ) in some archaeal species relies on recognition of broken ends by Ku which brings broken ends together, where exonuclease activity produces complementary ends for conclusion of DNA repair. The proteins that mediate NHEJ in many archaeal clades have not yet been defined.

Microhomology-Mediated End Joining (MMEJ) (Figure 3a) repair is dependent on short regions of close homology between sequences upstream and downstream of the DSB. These microhomologies are revealed by cellular exonucleases, allowing complementary sequences to anneal, producing a flapped substrate which is likely trimmed by flap endonuclease (Fen1) or the Rec J/GINS-associated nuclease (GAN) before DNA ligase seals the final nick(s) [25]. The unfortunate consequence of dependence on areas of microhomology is that they can sometimes be located far from the site of damage, and often intervening sequences are lost during repair [26]. Many details of archaeal MMEJ require additional studies, but DNA repair products consistent with MMEJ activities have been observed in both crenarchaea and euryarchaea when studying mechanisms of CRISPR–Cas immunity systems [27,28].

Non-Homologous End Joining (NHEJ) (Figure 3b) does not require large- or even micro-regions of homology for repair of DSBs. Instead, broken ends are brought together in a protein-mediated complex involving the DNA end-binding Ku protein and a multitude of likely dynamically associated DNA repair enzymes. Although the molecular details have not been determined, Ku bound ends are exonucleolytically processed to generate 3′ ends that can be extended by strand-displacement synthesis by DNA polymerase [29,30]. Synthesis by DNA polymerase bridges the DSB, allowing DNA ligase to seal resulting nicks. Archaeal NHEJ relies on exonuclease activity to produce a template for strand resynthesis and can thus, result small deletions of genetic information.

### 2.2. Homologous Recombination (HR)-DSB Repair

**Figure 4:**
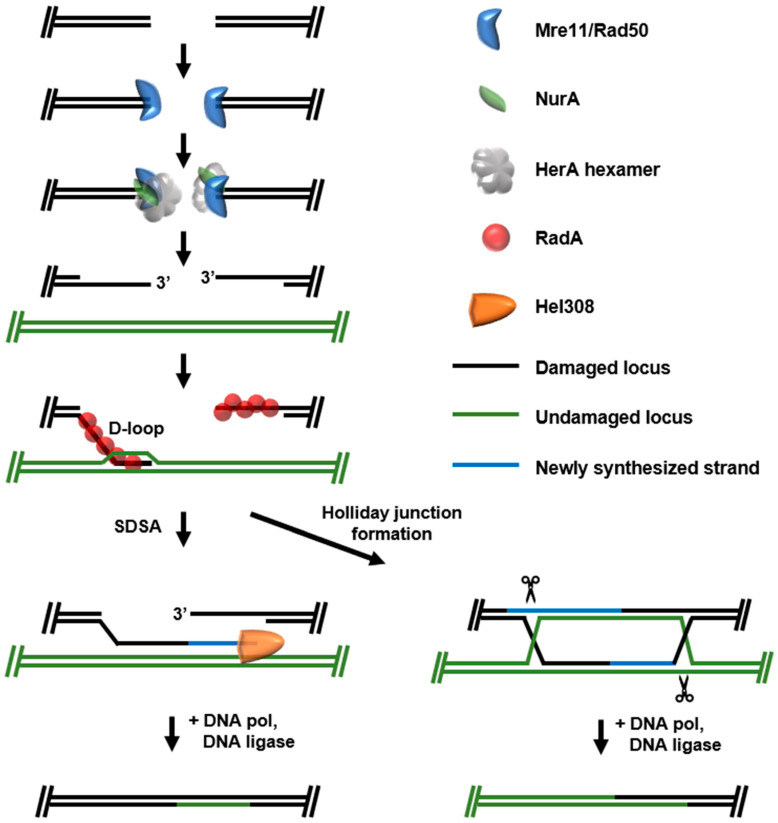
Homologous recombination-based DSB repair in Archaea. Broken end recognition by the Mre11/Rad50 complex allows formation of 3′ overhangs by the HerA hexamer. RadA forms a nucleoprotein filament on the 3′overhangs and facilitates initiated homologous recombination through strand invasion. In the case of just one strand invasion event, synthesis-dependent strand annealing (SDSA) can occur before repair conclusion, a non-crossover event. If both strands are involved in local strand invasion events, a Holliday junction may form, the resolution of which may lead to crossover events.

The retention of multiple, in some cases many tens of, genomes in some archaeal species facilitates a more accurate DSB repair mechanism, dependent on homologous recombination (HR-DSB) [31,32,33]. HR-mediated methods for repairing DSBs have a higher energetic cost but are generally error-free because an undamaged template strand is made available without the need for strand resectioning (Figure 4). HR-DSB is considered accurate but it is not without consequence, as crossover events or gene conversions are common results of HR—likely playing a significant role in the evolution of archaeal genomes. Recognition of DSB ends and subsequent “resectioning” by exonucleases to produce single stranded 3′ ends is initiated by the universally conserved Mre11/Rad50 complex (SbcC/SbcD in Bacteria) with resectioning activities performed by RecBCD [34,35,36,37]. Resectioning steps allowing formation of 3′ ssDNA overhangs in Archaea have historically been unclear, [38,39]. Mre11/Rad50 genes are commonly encoded in operons with both a bipolar helicase HerA and a novel nuclease NurA in hyperthermophilic archaea, implying a functional link of these three enzymes to drive resectioning activities [40,41]. Current models suggest HerA and NurA are responsible for activities that generate the 3′ ssDNA ends after recruitment by the DSB localized Mre11/Rad50 complex [42,43]. In *Sulfolobus*, HerA resectioning is required for cell viability with the functional HerA complex existing as a mixture of hexameric and heptameric states bound around strands of dsDNA. The nuclease NurA is thought to preferentially bind on the outside of the hexameric HerA–dsDNA substrate, where ATP-dependent helicase activity of the HerA ring is thought to stimulate NurA activity, likely by coupling translocation and ssDNA substrate presentation for NurA to degrade [44,45,46,47]. How this complex is specifically activated by Mre11/Rad50 after recognition of DSBs to produce appropriate resectioning remains elusive and is vitally important information for understanding the initiation of DSB repair by HR.

After resectioning, free ssDNA 3′ ends are recognized by the conserved recombinase RadA (bacterial RecA; eukaryotic Rad51) which polymerizes along the length of the ssDNA region [48,49,50,51]. The resulting dynamic RadA nucleoprotein filament then binds to local dsDNA and searches for a homologous sequence. Once located, the resulting intermediate structure is referred to as the “D-loop”, the primary initiation point for HR-DSB repair. D-loop formation permits two alternative and divergent pathways to complete repair. In some cases, only one 3′ end of the resectioned DSB is captured into a D-loop and is subsequently used as a starting point for DNA synthesis using the invaded, undamaged DNA strand as a template in a process termed synthesis-dependent strand annealing (SDSA) [52,53]. The newly synthesized strand is then unwound from the invaded stand, where it can anneal with homologous sequences on the other side of the DSB to accurately repair the lesion. Unwinding of the newly synthesized strand is facilitated by the helicase Hel308, which uses a winged-helix domain in a ratchet mechanism to translocate 3′-5′, simultaneously separating DNA strands in an ATP-dependent manner [54,55]. SDSA HR-DSBR does not result in a crossover event but can result in gene conversion if the invaded strand used as a template and the invading strand are heterozygous [56]. 

Alternatively, both ends of the DSB can be captured, giving rise to a Holliday junction. Once generated, the Holliday junction must be resolved before repair can be completed. The archaeal Holliday junction resolvase Hjc specifically recognizes four-way junctions of DNA and uses nuclease activity to resolve the junction [57,58]. The resultant newly formed junctions can have significant impacts on genomic integrity and Holliday junction resolution is likely an important point of regulation for HR-DSBR. The cleavage activity of Hjc is repressed by phosphorylation in *Sulfolobus islandicus*, which is consistent with bacterial and eukaryotic resolvases [59,60]; cells are more resistant to high doses of DNA damaging agents when the phosphomimetic version of Hjc is expressed [61].

How cells commit to an accurate or error-prone DSB repair pathway has significant consequences for gene conversion, genomic stability, and crossover events. Competition between the pathways is likely, and in halophilic Archaea, Mre11/Rad50 appears to influence rates of HR [62]. When mutations to the Mre11/Rad50 complex in *Haloferax volcanii* were introduced that were predicted to recruit resectioning enzymes essential for HR, instead of activating HR-DSBR, usage of HR-DSBR became “unrestrained”. These Mre11/Rad50 mutant strains appeared to grow faster but were more challenged by DNA damaging agents, suggesting correct regulation of both error-prone and HR pathways is required for optimal DNA repair. Notably, halophilic Archaea are generally polyploid, suggesting that species ploidy may not be completely accurate in determining whether HR-based DSBR methods are preferred. Post translational modification of DSB repair components, including methylation of the Mre11/Rad50 complex in *Sulfolobus acidocaldarius*, likely also contribute to the efficiency and rates of different DSBR pathways [63,64]. As DSBs are a likely consequence of replication apparatuses reaching nicks or damaged DNAs, it is perhaps not surprising that many of the DSBR enzymes maintain interactions with known components of the replicative apparatus. Hjc, Mre11/Rad50, and Hel308 are all known to interact with DNA replication proteins, reinforcing the link between double-strand break repair proteins and locating to areas of active replication [34,65,66].

### 2.3. New Resources Emerging from DSB Repair Pathways

As more molecular details of both HR and error-prone archaeal DSB repair mechanisms emerge, opportunities for practical molecular biology applications have arisen. The induced and natural competency of many archaeal species permit genetic manipulations, most dependent on HR-directed gene conversion and integrations of new DNA. The archaeal Hel308 enzyme, believed to be responsible for strand displacement during SDSA, has been extensively studied for use in nanopore sequencing [67]. DSB repair is also an essential process for CRISPR viral defense systems found in ~85% of Archaea, in which Cas enzymes generate guided double-strand breaks which are subsequently repaired by non-HR DSB repair pathways, NHEJ, and MMEJ [26]. Knowledge of conserved non-HR DSB repair has allowed for development of the first type II CRISPR–Cas-based genomic editing systems in archaea [27,68].

## 3. Mismatch Repair (MMR)

DNA polymerases must not only perform replication with physiologically relevant high speeds to avoid disruption of proper gene expression, but also with high fidelity. The necessity for fast DNA synthesis inevitably leads to errors by replicative DNAPs, with incorrect base incorporations once every 10^6^–10^10^ nucleotides under normal conditions. In general, misincorporating a purine for purine (or pyrimidine for pyrimidine) occurs more readily, resulting in transitions (A:T to/from G:C) rather than transversions (A:T to/from C:G) [69,70]. Failure to efficiently recognize and repair the resulting mismatches leads to increased mutation rates [71]. The canonical pathway of DNA mismatch repair (MMR) is the MutL/MutS/MutH pathway, which has been well characterized in Bacteria and Eukarya [72,73], but many Archaea do not encode obvious homologs of these enzymes. The apparent lack of MutL/MutS in many Archaea drove efforts to describe an alternative pathway for mismatched base recognition and resulted in identification of the novel NucS/EndoMS nuclease. Here, we summarize the MutL/MutS pathway and recent insights into potential Nucs/EndoMS-based MMR.

### 3.1. MutL/MutS

The MMR machinery in Bacteria is likely localized to nascent DNA strands during DNA replication, where mismatched bases are first recognized by MutS. Once bound to mismatched DNA, MutS subsequently recruits MutL, and the MutS–MutL complex can then stimulate the nuclease activity of MutH. MutH specifically nicks at unmethylated GATC methylation sites, allowing discrimination between the template and nascent DNA strands. Cutting at unmethylated GATC sites ensures the nick (and subsequent degradation of mismatched DNA) is performed on the newly synthesized strand, which likely contains the error. The helicase UvrD is then thought to perform strand displacement, with subsequent degradation of the damaged strand by generic cellular exonucleases. This allows DNA polymerase to resynthesize the resulting gap from the undamaged strand and DNA ligase to seal the nick. Eukaryotic MMR is similar but does not contain MutH or UvrD [74,75]. It is instead thought that asymmetric loading of MutS/MutL, mediated by interactions with replisome components, directs MutL nuclease activity to the newly synthesized strand. The eukaryotic repair polymerase contains both replication and exonuclease activities, which are believed to facilitate removal and degradation of the damaged strand during resynthesis.

Studies of methanogenic Archaea which encode MutS/MutL homologs indicate that the initial steps of this pathway are likely comparable to eukaryotic-like MMR. *Methanosaeta thermophila* MutS1 binds mismatched dsDNA but has low affinity for perfectly matched duplexes. The corresponding archaeal MutL makes single stranded nicks at the site of mismatches, which are assumed to be directed to a specific strand in a similar manner to eukaryotic homologs [76]. The importance of MutS/MutL for MMR, however, does not seem to be ubiquitous, as homologs from halophilic Archaea are readily deleted with no apparent effect on mutation rate [77], and coupled with the apparent lack of MutH in Archaea, suggested an alternative MMR pathway was present in these species.

### 3.2. NucS/EndoMS

To identify potential MMR enzymes in species apparently lacking MutL/MutS, cosmid-expressed Pyrococcus furiosus genome regions were screened for the ability to cleave the DNA backbone at the site of mismatches and resulted in the isolation of NucS/EndoMS [78]. NucS (later aliased EndoMS) constitutes a novel family of archaeal and bacterial endonucleases originally identified when bioinformatically screening the genome of Pyrococcus abyssii for an amino acid motif known to bind the replication clamp PCNA [79]. Initially, NucS/EndoMS was investigated for activity upon branched structures which arise due to DNA damage or as a replication intermediate, but not for recognition of mismatched bases [80]. However, deletion of NucS/EndoMS in Mycobacterium smegmatis and Corynebacterium glutamicum resulted in an increased mutation rate with mutations that match transitions (the expected result of MMR deficiency), suggesting an in vivo MMR role [81].

**Figure 5:**
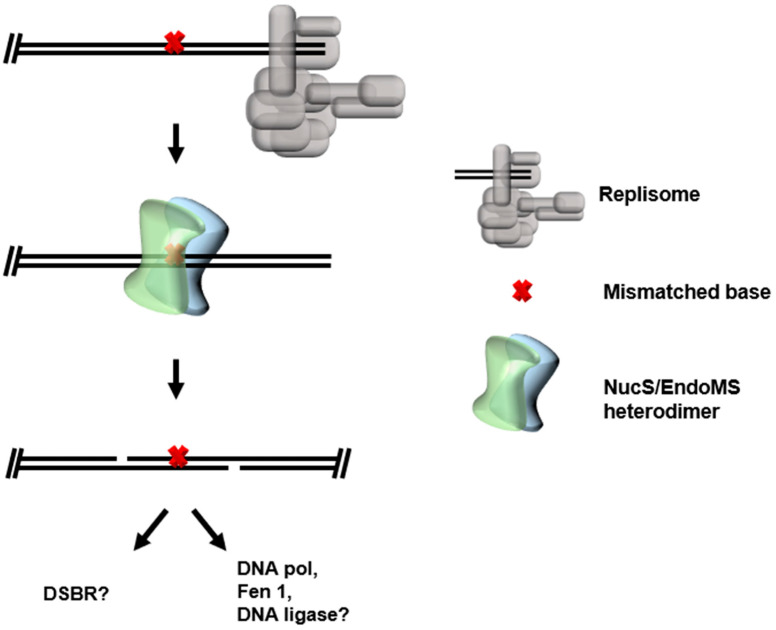
Potential mismatch repair pathways through NucS/EndoMS. NucS/EndoMS may surveil newly synthesized areas of the genome for mismatch incorporations. If a dual cut is made as in vitro, a DSB-like substrate would be formed, requiring DSB repair pathways or more immediate repair conclusion by DNA Polymerase, Flap endonuclease, and DNA ligase.

Biochemical and structural characterizations of archaeal NucS/EndoMS revealed MMR-like activities differing significantly from the Mut enzymes, offering dual activities of mismatch recognition and backbone cleavage in a single enzyme. Apo- and mismatched DNA:NucS/EndoMS complex structures suggest the enzyme forms a functional dimer and uses a “base-flipping” mechanism used by many DNA glycosylases (re Base Excision Repair) for recognition of mismatched bases [82,83,84]. Strikingly, offset cuts are made on opposite strands in vitro by NucS/EndoMS after mismatch recognition, resulting in a substrate akin to a DSB with two 4 nt 5′ overhangs [70,85]. If this activity is maintained in vivo, the potentially cytotoxic consequences of DSBs (re DSBR in Archaea) must then be dealt with—a seemingly disadvantageous way of dealing with a simple mismatched base. The activity of NucS/EndoMS is likely directed or modulated in vivo through interactions with the replisome. Both bacterial and archaeal NucS/EndoMS interact with their respective replisome clamp domains (the bacterial beta-clamp and archaeal PCNA). The interaction between the beta-clamp and NucS/EndoMS is required for efficient nuclease activity and high-fidelity replication in Bacteria [70]. Additionally, activity upon branched substrates by archaeal NucS/EndoMS is enhanced when in complex with PCNA [86]. Taken together, it is likely that EndoMS makes contacts with the replisome, allowing mismatches to be quickly recognized after DNA synthesis, and aiding in resolution of aberrant forked substrates. However, if NucS/EndoMS-mediated MMR still produces dual cuts of DNA in the cell, the downstream consequences of the resultant DSB-like product remain unclear (Figure 5). HR-based DSB repair is a probable compliment pathway, but this is less likely in Archaea, which spend significant time in a monoploid or diploid state such as the *Sulfolobales* and certain methanogens [87]. Furthermore, there remain Archaea without characterized MutL/MutS or EndoMS enzymes, suggesting undiscovered avenues for MMR in these species.

## 4. Ribonucleotide Excision Repair (RER)

Purposefully incorporated ribonucleotides are common, i.e., RNA primers for DNA replication, and it is posited that many rNTP incorporation events by DNA polymerases are evolutionarily conserved [88]. However, the lack of efficient removal of ribonucleotide monophosphates (rNMPs) has detrimental effects on genome stability, specifically by altering DNA form and enhancing hydrolytic activity brought by the 2′OH, which is lacking in dNTPs [89]. During DNA replication, DNA polymerase must not only reduce mismatches by distinguishing between DNA bases but must also monitor the usage of dNTPs vs. rNTPs [88]. Cellular concentrations of rNTPs can be magnitudes higher than that of dNTPs, and thus, inappropriate incorporations of rNTPs into dsDNA are inevitable. Archaeal D family DNA polymerases have been shown to incorporate 1 rNTP every ~1000 bases, and archaeal B family DNA polymerases every ~2500 bases [90,91].

**Figure 6:**
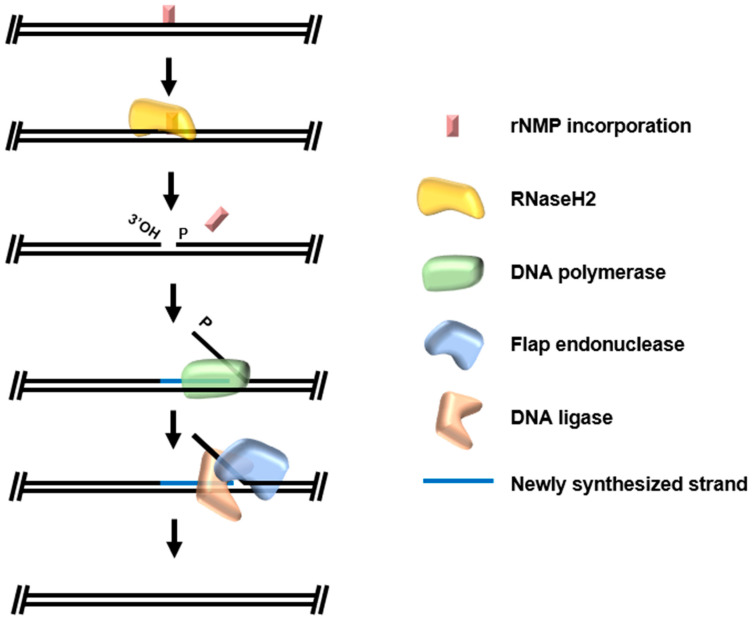
Archaeal ribonucleotide excision repair. Embedded ribonucleotide monophosphates (rNMPs) are recognized and specifically excised by RNaseH2, resulting in one nucleotide gap with 3′-hydroxyl. Repair is concluded when DNA polymerase performs strand-displacement synthesis and the activities of flap endonuclease and DNA ligase remove the original strand and seal the resulting nick.

Ribonucleotide excision repair (RER) is the universally conserved pathway for removal of rNMPs incorporated into dsDNA. RER is initiated by RNaseH2, generating a nick to the 5′ of the embedded rNMP. In Eukarya, the 3′ end generated by this nick is displaced by synthesis by DNA polymerase δ/ε, and the resulting flap (with embedded rNMP) is cleaved by flap exonuclease Fen1 [92]. In Bacteria, the strand displacement synthesis and flap cleavage are both carried out by DNA polymerase I [93]. Archaeal RER activities were tracked in *Thermococcus kodakarensis* lysates lacking computationally annotated homologs of RER enzymes on dsDNA substrates with a single embedded rNMP [90]. An archaeal RNaseH2 homolog recognizes the rNMP incorporation and nicks at the 5′ end, allowing strand displacement synthesis by the family B DNA Polymerase (Pol B) repair DNA polymerase in *Thermococcus kodakarensis* (Figure 6). Consistent with a eukaryotic-like RER mechanism, the repair polymerase Pol B does not have flap exonuclease activity, relying instead on the flap endonuclease activity of Fen 1. Once the flap is cleaved by Fen1, the resulting DNA nick is ligated by DNA ligase and the newly synthesized strand is free of embedded ribonucleotides.

Embedded single ribonucleotides are sometimes caused by inefficient Okazaki fragment maturation—the removal of lagging strand RNA primers during DNA replication [94]. In *Thermococcus*, Okazaki fragment maturation resembles RER as it relies upon the strand displacement activities of Pol B, RNaseH2, Fen1, and DNA ligase [95]. In some cases, the 5′-3′ exonuclease activity of GAN is used to remove the displaced RNA flap in lieu of Fen1, but in the absence of GAN, the RER enzymes Fen1 and RNaseH2 are reported to function together to remove the displaced RNA flap [95,96]. Cells need either GAN or both Fen1/RNaseH2 for survival, not only suggesting that RER is possibly sufficient for Okazaki fragment maturation, but also the activity of GAN exonuclease during DNA replication is sufficient to maintain viable levels of rNTP:dNTP in cellular DNA. *Thermococcales* are generally polyploid, raising the possibility that increased homologous recombination events often expose DNA strands to enzymes responsible for maintaining genomic maintenance—allowing retained DNA repair pathways to crosstalk and potentially compensate for deficiencies of any individual repair pathway.

## 5. Base Excision Repair (BER)

Not all DNA damage arises from mistakes made by cellular machineries—in fact, single base loss (apurinic/apyrimidinic (AP) sites) and modifications (i.e., alkylation, deamination, and oxidation) are the most common DNA damage. If left unrepaired, these modifications are correlated with high mutation rates incompatible with sustained life [97,98,99], and the propensity for such damages may be raised by the external environments of the cell. Archaea often occupy niche and extreme environments which increase the prevalence of exogenous damage sources but nonetheless utilize the universally conserved base excision repair (BER) pathway to remove damage from DNA [100]. The canonical BER pathway involves recognition of specific base modifications by a glycosylase appropriate to each modification; a single glycosylase can recognize multiple modification types. Glycosylases can act as monofunctional or bifunctional enzymes. Monofunctional glycosylases simply cleave the glycosidic bond between the base and phosphodiester backbone, “base excision”, leaving an AP site. AP-specific lyases then cleave the DNA backbone to create a 5′ deoxyribosephosphate (dRP) and 3′ unsaturated aldehyde (UA) moiety via β-elimination or 3′ phosphate via β/δ-elimination [101]. Bifunctional glycosylases exhibit both base excision and AP lyase activity. After glycosylase activity, AP nucleases convert the 3′ UA or phosphate into a free 3′OH for extension by DNA polymerase. DNA polymerase may incorporate just one nucleotide (short-patch BER) or multiple nucleotides (long-patch BER). In short-patch BER, dRP-lyase activity of DNA polymerase removes the 5′ deoxyribosephosphate while resynthesizing a single base, leaving a nick which may be sealed by DNA ligase. In long-patch BER, strand displacement activity of DNA polymerase displaces the 5′ deoxyribosephosphate containing the strand as multiple bases are incorporated. The damaged strand can then be removed at a junction site by Flap endonuclease, and the resulting nick sealed by DNA ligase.

**Figure 7:**
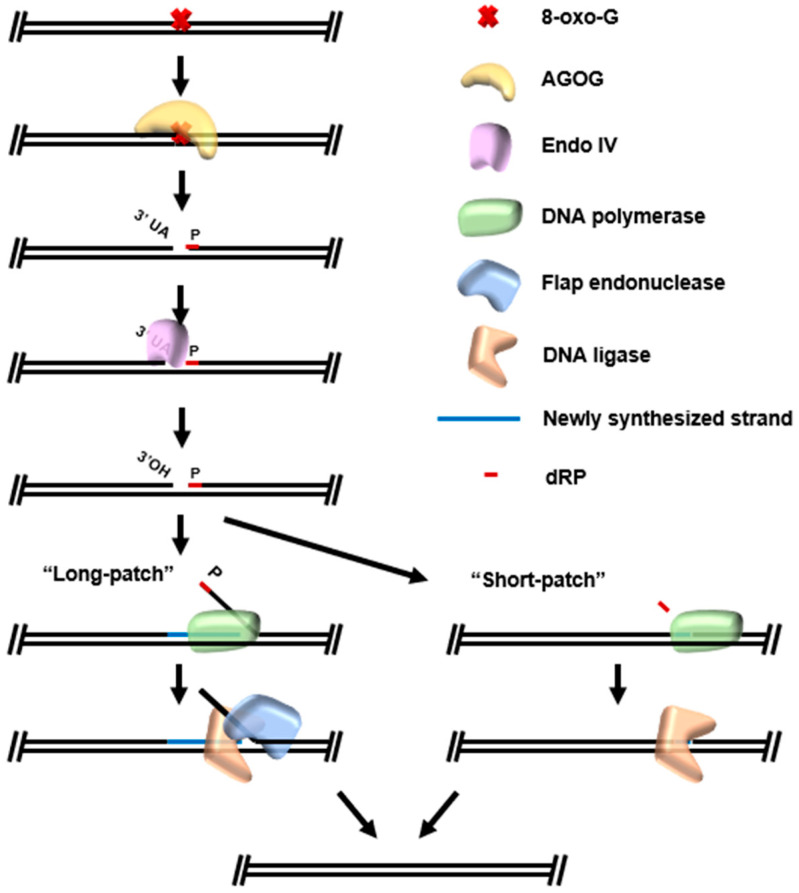
Reconstituted archaeal base excision repair from *Thermococcus kodakarensis*. AGOG recognizes 8-oxo-G modifications and acts as a *bifunctional* glycosylase, both excising the damaged base and cleaving the DNA backbone at the site of damage. The resulting substrate contains a 3′ unsaturated aldehyde (UA) and 5′ dRP. Damage repair is initiated by the activity of Endonuclease IV, which converts the 3′-UA to an extendable 3′-hydroxyl group. In long-patch base excision repair (BER), strand displacement activity of DNA polymerase during synthesis is used in tandem with flap endonuclease and DNA ligase to conclude repair. In short-patch BER, dRP lyase activity intrinsic to DNA polymerase simply removes the dRP moiety while synthesizing the correct base from the undamaged strand, and DNA ligase seals the nick.

Archaeal BER has been completely reconstituted in vitro (Figure 7). The Ogg-subfamily archaeal GO glycosylase (AGOG) of *Thermococcus kodakarensis* [102] recognizes 8-oxo-guanine (8oxoG) modifications which result from the oxidation of guanine and acts as a bifunctional glycosylase to perform base excision and cleave the DNA backbone. The activity of AGOG-like BER enzymes leave a 1 nt gap with a 5′ phosphate and 3′ UA after recognition of a chemically modified base. The 3′ unsaturated aldehyde must be chemically converted to a 3′OH by an AP endonuclease (Endo IV in *T. kodakarensis*) before strand-displacement synthesis by Pol B, flap cleavage by Fen1, and ligation of the resultant nick by DNA ligase. Structural studies of AGOG have also provided insight into the structural basis of specificity, determining recognition and cleavage of damaged substrates by AGOG is mediated by a conserved proline and phenylalanine motif allowing appropriate conformational freedom [103,104,105].

Recognition of chemically modified DNA in Archaea has also been shown to be mediated directly by DNA backbone cleavage activity of endonucleases rather than glycosylases. In hyperthermophilic archaeal species, where temperature-dependent chemical modifications are presumably more common, the existence of multiple damage repair initiating enzymes is likely advantageous. Alternative excision repair (AER) pathways do not rely on the excision of the damaged base and subsequent AP site recognition as separate steps, potentially accelerating the repair of specific damage types. Endonuclease V has been shown to recognize all deaminated bases in *Ferroplasma acidarmanus*, and specifically hypoxanthine (deaminated adenine) bases in *Pyrococcus furiosus* and *Thermococcus barophilus* [106]. Endonuclease V binds and cuts two nucleotides away from the 3′ end of the deaminated base, initiating downstream repair processes. Another novel nuclease, Endonuclease Q (EndoQ), was recently discovered in *P. furiosus* and cleaves the DNA backbone at deaminated bases, oxidized bases, and AP sites [107,108]. Similar to the MMR enzyme EndoMS and most glycosylases, EndoQ uses a “base-flipping” mechanism, placing bases in an active site adjacent pocket which allows for cleavage in the event of improper base pairing resulting from oxidized bases such as 5-hydroxyuracil and 5-hydroxycytosine. The wide substrate range of EndoQ, coupled with its studied interactions with PCNA, suggest that EndoQ may also localize to newly synthesized DNA, recognizing DNA damages that may not lead to misincorporations by DNA polymerase.

### New Resources Emerging from BER Pathways

The characterization of a large selection of archaeal BER enzymes specific to particular damage types has provided an advantageous protocol for biochemical analyses of DNA damages on a genome-wide scale. In RADAR-seq [109], enzymes specific to a DNA damage type are used to make lesion-dependent nicks on sequencing libraries prepared from purified genomic DNA. DNA repair enzymes which create an extendable 3′OH at the nick site are then utilized, followed by strand-displacement synthesis by DNA polymerase in the presence of methylated dNTPs. Methylated bases are thus incorporated into the site of DNA damage repair and can be detected via PacBio SMRT sequencing [110], allowing genome-wide coverage of the locations of a single DNA-damage type. RADAR-seq has been used to exhibit the increase in genomic rNTP incorporation after deletion of RNaseH2—which is essential in rNTP removal—and will likely continue to be established as an accepted method of assessing genome-wide DNA damage.

## 6. Global Genomic Nucleotide Excision Repair (GG-NER)

**Figure 8:**
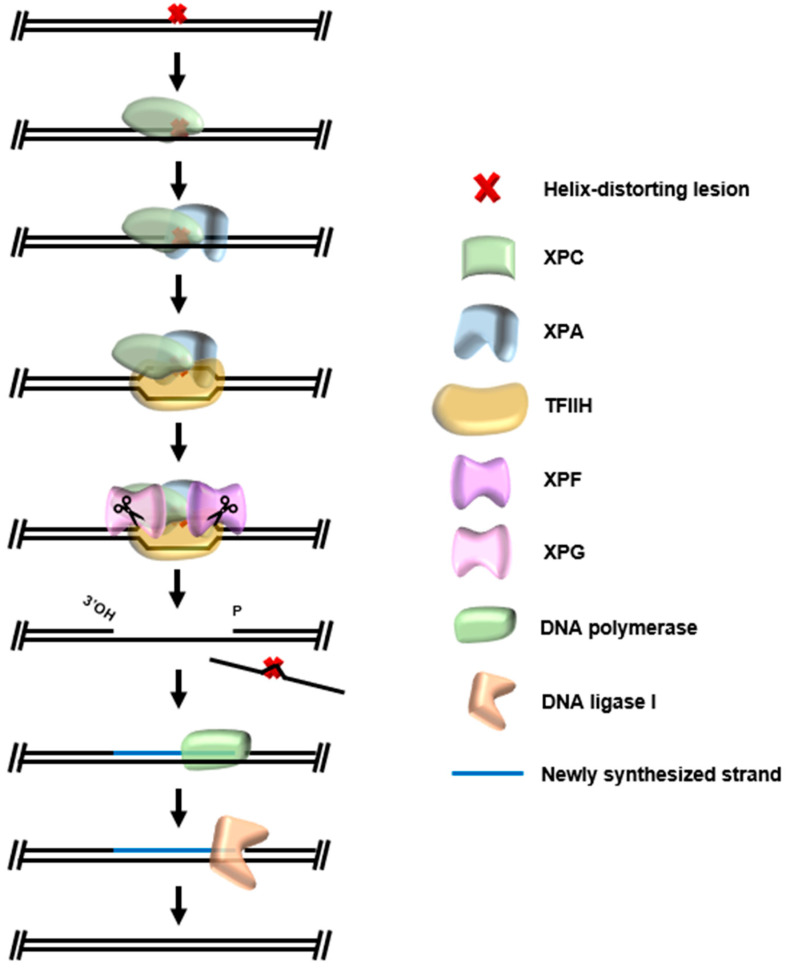
Eukaryotic global genomic nucleotide excision repair. DNA damages which distort the DNA double helix are recognized by XPC, which recruits the damage recognition XPA and TFIIH complex. Components of the TFIIH complex melt strands of DNA around a verified DNA lesion, allowing cuts of the damaged strand by XPG and XPF. The TFIIH complex uses helicase activity to “excise” the damaged strand, allowing conclusion of repair by DNA polymerase and DNA ligase I.

Some DNA damages, i.e., UV-induced photoproducts, result in a distortion of the dsDNA helix which has stalling effects on critical processes such as replication and transcription. DNA repair mechanisms have evolved to detect the general distortions of the DNA backbone rather than the actual modification, which allows detection at a broad range of DNA lesions. Global genomic nucleotide excision repair (GG-NER) in Bacteria and Eukarya relies on enzymes to recognize the “bulky lesion” and direct strand-specific cuts on the damaged DNA strand [111,112]. The DNA damage, now between two nicks, is thus primed for “excision” from the DNA allowing resynthesis from the undamaged strand, and nick ligation to complete repair. In Bacteria, NER is mediated by the UvrA2/B/C/D enzymes. Helix distortions are first recognized by the UvrA dimer and damage is subsequently verified by UvrB [113,114]. The activity of UvrB converts general strand distortion detection by UvrA into damage- and strand-specific detection, which directs the nuclease activity of UvrC either side of the DNA damage [112,115]. The UvrD helicase can then excise the damage containing strand from the genome, allowing for strand resynthesis by DNA polymerase I and nick sealing by DNA ligase. The core steps of eukaryotic NER resemble a slightly more sophisticated bacterial NER (Figure 8). DNA helix distortions are first recognized by the XPC repair protein, and then damage is verified by the XPA protein to form a pre-incision complex. Helicases XPB and XPD then separate DNA strands at the site of damage; the orientation of the resulting complex allows strand-specific cuts by XPG and XPF on either side of the site of DNA damage [112,115,116]. The damage containing strand is excised in complex with TFIIH [117], allowing strand resynthesis by DNA polymerase δ or ε, and nick sealing by DNA ligase I [118].

Some Archaea encode homologs of bacterial Uvr proteins which appear to be active in bulky DNA lesion removal, i.e., deletion of UvrA, UvrB, and UvrC in *Halobacterium* result in significant UV sensitivity phenotypes [119,120]. The majority of Archaea, however, encode homologs of critical eukaryotic NER proteins, in particular helicases XPB/XPD and endonuclease XPF (Figure 1) [121]. No NER pathway, however, has yet been explicitly defined in Archaea, with research focusing on drawing parallels from individual enzymes conserved between Eukarya and Archaea. Such enzymes tend to exist outside the context of a multi-protein complex, allowing for ease of purification and crystallization. For example, independent structures of archaeal XPD, normally a component of the multienzyme TFIIH complex in eukaryotes, from *Thermoplasma acidophilum* and *Sulfolobus acidocaldarius* revealed a distinct four domain structure; disease-causing mutants from human XPD could be mapped to functionally critical sites of the structures [117,122,123]. 

While research into archaeal XP homologs has been structurally fruitful, establishing the NER pathway in Archaea has remained challenging and elusive. Perturbations in the UvrA, UvrB, and UvrC homologs found in Halobacterium resulted in almost total loss of resistance to UV exposure, but it remains unseen if these homologs function in a recognized NER pathway, and the Uvr proteins are only found in a minority of Archaea [119]. Conversely, deletions of XPB, XPD, and XPF from *Thermococcus kodakarensis* resulted in only slight sensitivity to moderate doses of UV irradiation [124], suggesting these enzymes are involved in—but are not required for—UV damage response. Additional factors could potentially play a role in archaeal NER, and in some cases, the eukaryotic-like NER enzymes are paired with auxiliary nucleases. XPB helicase is sometimes found encoded in an operon with a nuclease named Bax1 and these enzyme act in concert to open a DNA bubble and make cuts [125]. In many XPF-encoding species, the 3′-5′ exonuclease HAN is often encoded, potentially recapitulating in vitro experiments where XPF and HAN form a functional nuclease complex [126]. Recent biochemical examinations of archaeal XPF, or Hef, have investigated the enzyme in the context of replication restart and Holliday junction formation [120,127,128], but it is possible that XPF performs multiple functions within the cell—including one in an NER pathway.

The lack of direct evidence for NER in most Archaea has led to speculation that there is no conserved NER pathway in the domain and this deficiency is simply compensated for by increased activity of repair enzymes during stalled replisome restart after DNA polymerase is stalled by helix distortions. If a conserved NER pathway involving eukaryotic-like enzymes exists in Archaea, there remain several unrevealed details and it likely differs significantly from the eukaryotic pathway. Eukaryotic-like nucleotide excision involves strand nicking by two distinct exonucleases, XPF and XPG, but the latter is not found in Archaea. It is feasible that XPF is responsible for both cuts or it only makes one cut in an MMR-like mechanism. However, of prime importance is the question of damage recognition as there are no known homologs of the eukaryotic NER damage recognition enzyme XPC in Archaea. Thus, elucidating how bulky helix-distorting lesions are detected will be of great value in establishing archaeal NER.

## 7. Transcription Coupled Nucleotide Excision Repair (TC-NER)

In Bacteria and Eukarya, NER can be initiated by recognition of transcription elongation complexes (TECs) which stall upon DNA lesions entering the active site of RNA polymerase (RNAP) during transcription, a process termed transcription coupled DNA repair (TCR). Utilizing actively transcribing RNAPs to sense DNA damage offers an evolutionary advantage as actively transcribed regions of the genome are actively monitored for lesions. Akin to global NER, TCR has yet to be described in Archaea but current evidence suggests it is an active pathway in some clades. While studies in crenarchaea have revealed no significant change in DNA repair of transcribed versus non-transcribed strands [129], euryarchaeal species have displayed preferential repair of transcribed DNA strands—a hallmark of TCR [130]. Additionally, the archaeal RNAP—which closely resembles eukaryotic RNAPII—has been shown to stall specifically at template strand DNA damage [131].

**Figure 9:**
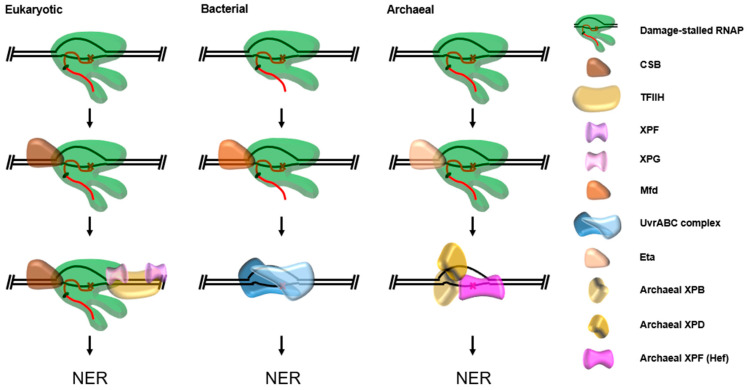
Current eukaryotic and bacterial models of transcription coupled nucleotide excision repair (TC-NER) and a hypothetical archaeal model. In all cases, RNA polymerase (RNAP) is arrested at template-strand DNA damage and recognized by the TRCF-CSB in Eukarya, Mfd in Bacteria, and potentially Eta in Archaea. The TRCF either backtracks RNAP or terminates transcription while recruiting NER enzymes directly to the site of damage. Homologs of the eukaryotic XP proteins found in many Archaea act in our archaeal model.

In eukaryotes, the CSB protein acts as the transcription repair coupling factor (TRCF), initially recognizing stalled TECs and allowing localization of TFIIH and other NER enzymes directly to the site of damage [132]. In Bacteria, the transcription termination factor Mfd acts as the TRCF, simultaneously recruiting the Uvr family of NER enzymes and terminating transcription to prevent the formation of mutant transcripts [133,134,135]. There are no homologs of either CSB or Mfd found in the archaeal domain, suggesting a potential archaeal TCR pathway—and TRCF—evolved separately. Recently, the first enzyme with transcription termination activity was reported in Archaea, euryarchaeal termination activity (Eta), and is intimately linked with other nucleic acid metabolic pathways and is a candidate for acting as the archaeal TRCF [136,137]. Euryarchaeal termination activity (Eta) requires DNA sequences upstream of RNAP, aids backtracked RNAPs, is ATP-dependent, and is non-competitive with elongation—all attributes shared with the bacterial TRCF Mfd. The eukaryotic TRCF, CSB, also requires DNA sequences upstream of a stalled RNAP. Mfd catches up to backtracked or stalled polymerases by “autonomously” patrolling DNA upstream of TECs [138]. Deletion of Mfd in Bacteria and Eta in Archaea produce a UV sensitivity phenotype, further suggesting they share an analogous role [136,139]. However, species which encode Eta also encode eukaryotic XP NER enzymes which have yet to be implicated in an NER pathway. Without an obvious damage recognition NER enzyme encoded, it is attractive to think of damage stalled RNAP fulfilling this role. If Eta acts as an archaeal TRCF analogous to Mfd, but recruits eukaryotic-like NER enzymes, another intriguing example of an archaeal physiological pathway with both bacterial and eukaryotic-like elements would be presented (Figure 9) and explicitly evidence TCR as a universally conserved DNA repair pathway for the first time.

## 8. Discussion

Archaeal DNA repair-based research has offered inspiring mechanistic insight into the strategies of preserving DNA stability in extremes once thought inhospitable. Surprisingly, such strategies are not unique and resemble those found in mesophilic Eukarya and Bacteria. How then, do extremophilic species maintain low mutation rates in extreme conditions using the “same tools”? Clues may lie in the apparent crosstalk of archaeal DNA repair pathways or their intimate links with replisome components, or perhaps extremophilic species successfully protect their genomes, avoiding DNA damages to begin with. At face value, strategies of recognition of DNA damages and their preparation for the core resynthesis machinery (i.e., DNAP, Fen1, DNA Ligase) are intrinsically fascinating, but perhaps the most alluring facet of archaeal DNA repair has recently been the development of new techniques at the protein and whole-genome level as archaeal species have become more genetically accessible. Novel archaeal DNA repair enzymes will likely continue to be characterized and find new roles in the exponentially growing biotechnology world. Bioinformatic approaches, such as RADAR-seq, will continue to provide population-/genomic-level DNA repair activities, and the continuously developing knowledge of DSBR in relation to CRISPR systems will surely yield more tools for geneticists. Super-resolution microscopy, once thought over encumbered by the small size of archaeal cells, has recently become optimized and used to image foci of DSB sites in *H. volcanii* [140], offering the DNA repair research as a platform for development of more broadly applicable procedures. The continued development of these (and new) technologies, however, will only be progressed alongside our understanding of archaeal DNA repair as a whole—and thus, identifying and answering the most pressing questions in the field must remain a priority.

Once thought a detriment to cellular health, the double-strand break is appearing more of an essential intermediate to many metabolic processes outside of replication, potentially altering our view of archaeal metabolic biology. How Archaea deal with such an intermediate has been resolved through multiple pathways (NHEJ, MMEJ, HR), but the next challenge is understanding how cells “decide” which of these pathways is most appropriate in a given context, and if the ploidy state influences rate of HR. One such context may be resultant DSBs from EndoMS activity during archaeal MMR which, if verified, will allow us to probe how cells use DSB substrates purposefully outside of the replisome. Generating depth to our current understanding is of great importance—but there still remain significant “unknowns” in the field which have yet to be resolved. Are there alternative pathways for MMR or BER yet undiscovered—and can recognition enzymes be repurposed? Does NER or the transcription coupled sub-pathway (TCR) exist in Archaea, and do they more closely mirror a prokaryotic or eukaryotic system? Finally, as interconnectedness and crosstalk between repair and replication systems becomes more apparent, how are repair pathways regulated, segregated, or organized in the context of the prokaryotic cell? The answers to these questions will not only provide a clearer picture of DNA maintenance in extremis, but likely hold intriguing insights into our own ancestral metabolic history.
